# The Protective Role of Bark and Bark Fibers of the Giant Sequoia (*Sequoiadendron giganteum*) during High-Energy Impacts

**DOI:** 10.3390/ijms21093355

**Published:** 2020-05-09

**Authors:** Georg Bold, Max Langer, Laura Börnert, Thomas Speck

**Affiliations:** 1 Plant Biomechanics Group Freiburg, Botanic Garden of the University of Freiburg, University of Freiburg, Schänzlestraße 1, D-79104 Freiburg, Germany; georg.bold@biologie.uni-freiburg.de (G.B.); max.langer@biologie.uni-freiburg.de (M.L.); laura.boernert@gmx.de (L.B.); 2 FIT - Freiburg Center for Interactive Materials and Bioinspired Technologies, University of Freiburg (FIT), Georges-Köhler-Allee 105, D-79110 Freiburg, Germany; 3 Cluster of Excellence livMatS @ FIT, Georges-Köhler-Allee 105, D-79110 Freiburg, Germany

**Keywords:** tree bark, energy dissipation, impact protection, fibers, hierarchical level, quasistatic compression, drop-weight test, microtensile test

## Abstract

The influences of (1) a high fiber content, (2) the arrangement of fibers in fiber groups, and (3) a layered hierarchical composition of the bark of the giant sequoia (*Sequoiadendron giganteum*) on its energy dissipation capability are analyzed and discussed regarding the relevance for an application in bioinspired components in civil engineering. The giant sequoia is native to the Sierra Nevada (USA), a region with regular rockfalls. It is thus regularly exposed to high-energy impacts, with its bark playing a major protective role, as can be seen in the wild and has been proven in laboratory experiments. The authors quantify the fundamental biomechanical properties of the bark at various length scales, taking into account its hierarchical setup ranging from the integral level (whole bark) down to single bark fibers. Microtensile tests on single fibers and fiber pairs give insights into the properties of single fibers as well as the benefits of the strong longitudinal interconnection between single fibers arranged in pairs. Going beyond the level of single fibers or fiber pairs, towards the integral level, quasistatic compression tests and dynamic impact tests are performed on samples comprising the whole bark (inner and outer bark). These tests elucidate the deformation behavior under quasistatic compression and dynamic impact relevant for the high energy dissipation and impact-damping behavior of the bark. The remarkable energy dissipation capability of the bark at the abovementioned hierarchical levels are linked to the layered and fibrous structure of the bark structurally analyzed by thin sections and SEM and µCT scans.

## 1. Introduction

The bark, the total of all tissues outside of the vascular cambium, represents the outermost layer of stems of woody plants [1]. It may fulfil a multitude of different physiological and ecological functions such as photosynthesis [2], transport plus storage of various substances [3,4] and stemflow [5]. Additionally, owing to its peripheral position in a plant’s stem, it fulfills several protective functions. These protective functions include, among others, herbivore and pathogen resistance and can be subdivided into chemical and anatomical defense mechanisms. The chemical defense mechanisms are based on the release of chemicals such as phenols, terpenoids or alkaloids that are harmful to the potential invaders [6]. The anatomical defense, on the other hand, is based on the mere mechanical resistance of the more or less thick bark, thus representing a protective barrier. This resistance may be enhanced by the accumulation of lignified cells [7] like in the bark of the tree of heaven (*Ailanthus altissima* (Mill.) Swingle). Apart from herbivores and pathogens, the anatomical defense also involves protection from abiotic factors such as high temperatures during forest fires [8,9,10,11] and mechanical influences. One example of a tree species possessing a bark with an outstanding insulation capability during forest fires is the giant sequoia (*Sequoiadendron giganteum* Lindl.). As previously indicated, the high bark thickness and its loose structure with air inclusions are the reasons for this high heat insulation capability [12,13]. Typically, for trees, a lethal situation during forest fires occurs when the temperature of the vascular cambium increases above 60 °C [13,14]. The vascular cambium is located subjacent to the bark and is only a few cell layers thick. As this sensitive tissue is crucial for the formation of secondary xylem (wood) and phloem (bast)—and thus the bark—it is essential for the survival of the whole tree. This highlights the protective function of the tree bark, since it represents the only layer surrounding the cambium. At the same time, the inner and outer structure of the bark differs from species to species, resulting in differences concerning the protective potential.

The bark as a protective layer surrounding the cambium becomes important not only during forest fires, but also when the cambium needs to be protected from mechanical influences. Such mechanical influences can be represented, for example, by rockfall events, which occur regularly on the slopes of the Sierra Nevada [15], where the giant sequoia is native to. The capability of the giant sequoia bark to protect the cambium during such high-velocity impacts by dissipating high amounts of impact energy has been proven before [16,17]. This property becomes even more evident when being compared to tree species being native to regions with no regular rockfall events, like the tree of heaven. The differences in bark thickness and composition are most likely the reasons for the divergent energy dissipation capabilities of these two tree species. Thereby, the bark of the giant sequoia is prominent in comparison to other tree barks concerning, e.g., its high fiber content, pronounced inner and outer structuring of the bark and its high proportion of air-filled spaces resulting in a low bark density. Determining the importance of these aspects of the giant sequoia bark composition in the context of impact protection is one aim of the study presented here.

In the context of impact protection, technical foams are often mentioned and discussed in the literature. As will be demonstrated later, the deformation behavior of the giant sequoia bark when being compressed resembles in many ways that of elastomeric open-pore (also termed open-cell) foams. Without anticipating the results presented in this paper, the authors will address several aspects of technical foams and biological structures with comparable properties in the introduction, as this will become important in the following chapters.

Concerning technical open-pore elastomeric foams, three phases of deformation when being compressed can be distinguished and also identified in stress–strain diagrams [18]. In a first step, the deformation is linear elastic, until a strain of typically around 5%. Here, the pore/cell walls bend when the load is applied. In a second step, characterized by nonlinear elasticity, the cells buckle elastically. Typically, the stress remains at a nearly constant level (termed elastic collapse stress) during this step, thus forming a long flat plateau in the stress–strain diagram. Finally, in a third step, the cells collapse completely and the stress rises rapidly due to cell faces and edges being forced together (densification). Especially during the second phase, high amounts of energy are dissipated. This is exploited in foams for crash protection and energy-absorbing systems. However, also when not dealing with elastomeric foams, such a plateau can be present. In this case, the cells collapse plastically. Generally, in foams, energy can be dissipated by (a) plastic bending of the cell walls, (b) buckling of the cell walls, (c) fracture or (d) compression or flow of fluid, if a fluid is present. Depending on the material and the composition of a foam as well as on the strain and strain rate applied, the foam’s deformation behavior upon compression can be plastic, elastic, viscoelastic or a combination of those.

A comparison of biological structures with technical foams can already be found in the textbook on the mechanical behavior of plants, [19], in which apart from many of the general considerations being relevant to the present study, comparisons of plant tissues to technical foams can also be found. As a more specific example, the authors of [20] reported the mechanically similar behavior of certain closed-pore living plant tissues (in their case, the inner primary cortex of stems of the liana *Aristolochia macrophylla*) and closed-pore technical foam rubbers. Gibson and Ashby [18] compared the compaction behavior of cork with that of polystyrene foam. Both show a low collapse stress of the cells, so that the peak stress during impact is limited. By a progressive collapse of cell walls, large compressive strains are possible, and thus large amounts of energy are dissipated. Unlike these closed-pore systems, the authors of [21,22] examined the compaction behavior and impact-damping capability of the pomelo peel, an open-pore parenchymatous foam layer with liquid-filled struts. Driven by its capability to dissipate high amounts of kinetic energy during impact, the structure of the pomelo peel was replicated as silica [23]. Furthermore, it was adapted and transferred to bio-inspired metal–metal composites [24] and fiber-reinforced open-pore metal-foam-based components [25]. For the pomelo peel, it could be proven that all the seven hierarchical levels examined were markedly involved in energy dissipation, and the structures on several of these hierarchical levels were transferred to the metal-foam-based components. First, a gradient in intercellular space from the outside towards the inside in the thickest part of the peel (the mesocarp) was adapted and transferred into the metal foam. Secondly, lignified and branched vascular bundles present in the mesocarp inspired the implementation of directed fiber bundles into the technical aluminum alloy foam structures. Thirdly, the two-material structure of the biological cells of the pomelo peel (i.e., the cell wall and membranes as a semipermeable solid hull filled with a liquid phase) was transferred to the metallic foam as a dual-layered strut. A beneficial influence on the mechanical behavior, namely in a higher plateau stress and a more homogeneous deformation behavior, was found as a result of adapting the foam structure on the abovementioned hierarchical levels.

In addition to the parenchymatous foam-like pomelo peel, the macadamia seed shell was investigated in [25,26]. However, in this case, the main focus was placed on crack deflection within the shell as a mechanism of impact protection. Energy dissipation by crack propagation has been proven as being relevant also in case of the quasibrittle inner layer (endocarp) of the fruit wall of the coconut [17,27,28]. For these structures, the relevance of several hierarchical levels has also been pointed out when regarding the impact-damping capability. Like all the above-mentioned biological examples, the bark of the giant sequoia can also be seen as a material compound consisting of multiple biological tissues. However, in this case, none of the tissues relevant for energy dissipation are parenchymatous (like the pomelo peel) or of high density, consisting of a hard and tough compound of cells (like the macadamia seed shell and the coconut endocarp).

In the following sections, the composition of the giant sequoia bark from the cellular level to the integral level (whole bark) is elucidated, giving an overview of the test object. Following this, the energy dissipation capability and diverse mechanical properties of the bark are presented, starting with the whole bark and ranging down to single bark fibers. By this, the underlying principles of the bark’s high energy dissipation capability are presented and discussed step by step. Finally, we outline a potential transfer of the insights gained during the present study into energy-dissipating bio-inspired fibrous concrete and ceramic components for building construction.

## 2. Results

### 2.1. Composition of the S. giganteum Bark

**Figure 1:**
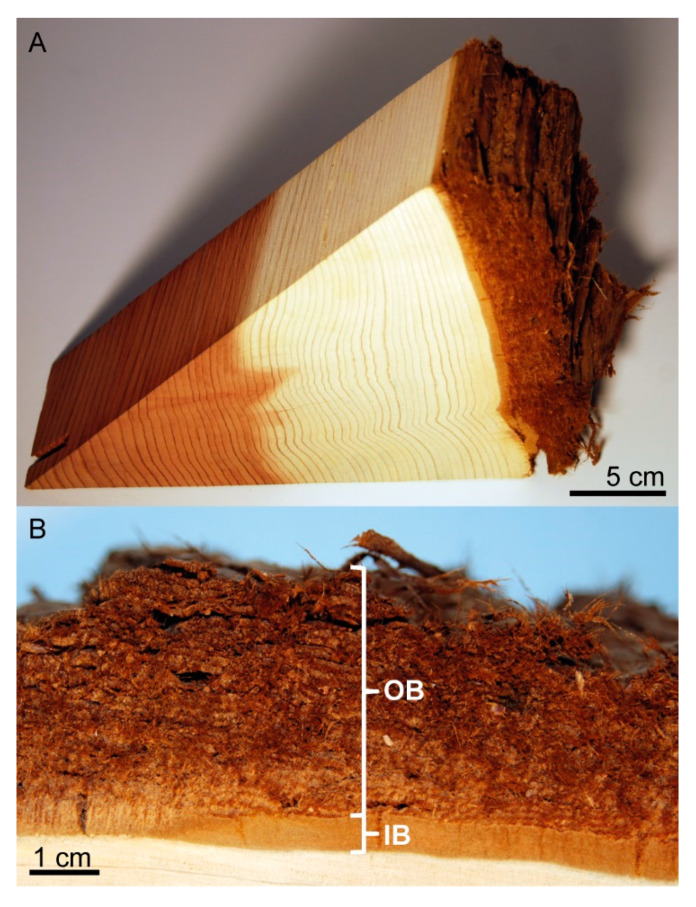
Sawed out stem segment of *Sequoiadendron giganteum*. (**A**) A segment of a tree cross section, including wood and bark. (**B**) In the bark of the segment, its subdivision into a dense inner bark (IB) and a less dense, highly structured and fibrous outer bark (OB) is visible.

The layered composition and the fibrous nature of the thick bark of *S. giganteum* is made up of a comparatively thin inner bark (bast), which is surrounded by a thick outer bark (rhytidome) (Figure 1). The inner bark has a higher density and is macroscopically markedly less structured than the outer bark. Long fibers that are arranged in layers become especially apparent in the outer bark. Primarily in the external part of the outer bark, platelets of the layered, fibrous bark spall off, resulting in a pronounced surface structuring.

**Figure 2:**
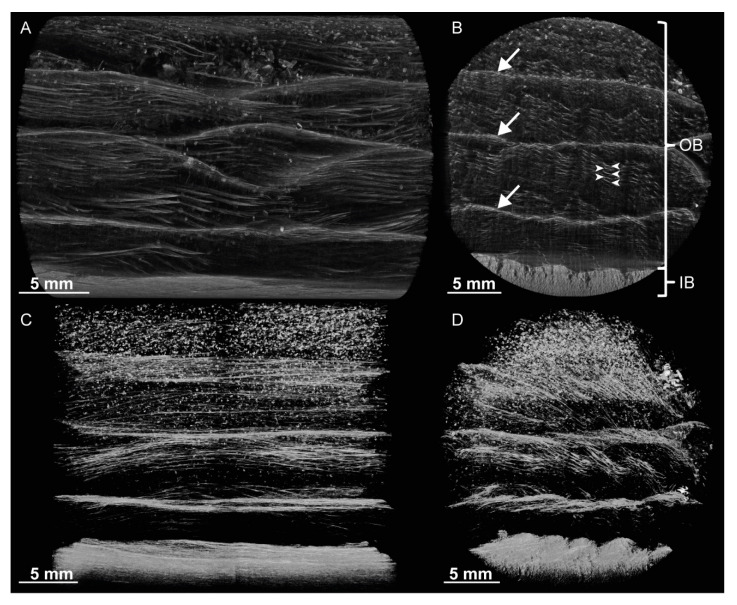
Images of a µCT scan of the bark of *S. giganteum*. (**A**) Radial section with partly intersecting layers (higher hierarchical level) of the outer bark. (**B**) Cross-section showing partly intersecting layers (higher hierarchical level) of the outer bark (IB = inner bark, OB = outer bark; arrowheads indicate representative layers on the lower hierarchical level, arrows indicate layers on the higher hierarchical level). (**C**,**D**) Segmentation of the µCT scan gives further insights into the layered structure of the outer bark, visualized by a radial sectional view (**C**) and a cross-sectional view (**D**). A video of the same µCT scan can be found in the Supplementary Materials.

µCT scans allow a closer view of the internal fine structuring of the bark (Figure 2) and allow—after segmentation of the µCT data—the visualization of the course of single fibers and their arrangement in layers. Layers occur on at least two hierarchical levels. The distance between layers on the lower hierarchical level typically amounts only to a few hundred micrometers (see arrowheads in Figure 2B), and these layers run almost parallel to each other. The layers on the higher hierarchical level are up to several millimeters apart from each other (see arrows in Figure 2B). They are characterized by a higher density than the in-between space and by a particularly high amount of fibers. Additionally, a three-dimensional network of the bark fibers in the outer bark occurs: the fiber layers of the higher hierarchical level do not exclusively run parallel to the bark surface but regularly intersect each other (Figure 2C,D).

**Figure 3:**
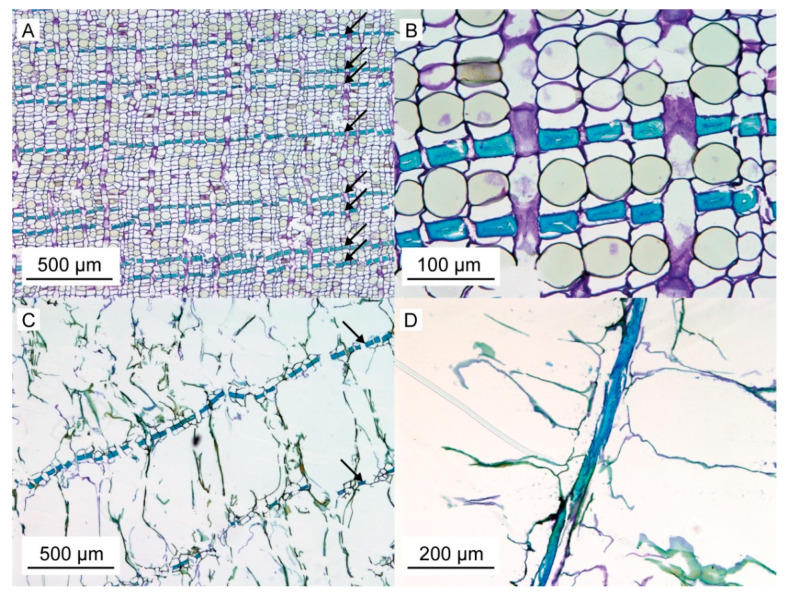
*S. giganteum* bark sections stained with toluidine blue. (**A**) Cross-section of the inner bark, already revealing layers of highly lignified, thick-walled fiber cells arranged in rows (stained blue, see arrows) located between thin-walled parenchymatous cells. (**B**) Enlarged view of a cross-section of the inner bark showing the nearly rectangular cross-section of the thick-walled fibers. (**C**) Cross-section of the outer bark with the largely intact thick-walled fibers still arranged in layers (arrows). As in the inner bark, many of these intact fibers might be interconnected via the short side of their cross-section, whereas the thin-walled parenchymatous cells between them are ruptured. (**D**) The fibrous nature of these thick-walled and elongated cells (stained blue) is visible in radial sections of the outer bark.

**Figure 4:**
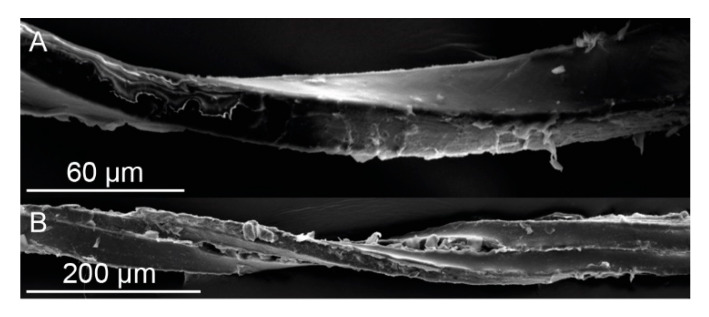
SEM images of a single extracted bark fiber (**A**) and two interconnected bark fibers (**B**) of the outer bark of *S. giganteum* showing the nearly rectangular cross-section of the fibers. An interconnection between fibers—if present—occurs exclusively between the short sides of the rectangular cross-section (cf. Figure 3). The twisting of the fibers is due to dehydration of the samples before and during SEM analysis.

Stained thin sections of the inner bark (Figure 3A,B) reveal the ontogenetic origin of the layered structuring of the outer bark. Microscopic analysis shows alternating layers of highly lignified, thick-walled fiber cells and thin-walled parenchymatous cells (Figure 3A). The cell walls of the highly lignified, thick-walled fiber cells fill up almost the entire cell, leaving almost no cell lumen (Figure 3B). These fibers are characterized by a nearly rectangular cross-section, and neighboring cells of the same type typically are attached to each other via the short side of this rectangle. Each layer of the thick-walled fibers comprises only one cell, whereas several layers of thin-walled parenchymatous cells are typically found between them. In general, this arrangement is present also in the outer bark (Figure 3C), with layers of thick-walled cells being often interconnected to their neighbors via the short side of their rectangular cross-section. However, in contrast to the inner bark, the parenchymatous cells located between these fiber layers are not intact any longer, but they are dried out and their cell walls are ruptured, resulting in a looser overall structuring. Radial sections of the outer bark reveal that the highly lignified, thick-walled fiber cells are highly elongated (Figure 3D) resulting in a fibrous structure that can be observed macroscopically. The nearly rectangular cross-section of the long fibers is also visible in SEM images (Figure 4A). Furthermore, the possible interconnection between adjacent fibers via the short side of the rectangular cross-section can be illustrated via SEM images (Figure 4B). Thereby, the interconnection forms at various spots along the fiber pair, resulting in a relatively strong, but not continuous, bonding between them. Therefore, fiber pairs can be defined as two (naturally) connected individual fibers. It appears that this pairing exists in both the outer and inner bark. This is particularly noticeable in the outer bark (Figure 3C), where fiber pairs or fiber triplets are often found although the rest of the cell structure has disintegrated.

In order to quantify the dimensions of the fibers’ rectangular cross-section, the fibers that were freshly prepared for the microtensile tests were measured. The dimensions of the fiber cross-section do not differ significantly between outer and inner bark. The longer side of the nearly rectangular fiber cross-section measures 40.7 ± 4.4 µm for the outer bark and 39.8 ± 4.8 µm for the inner bark (*n* = 10 each; compared via t-test, *p* = 0.668), and the shorter side measures 12.3 ± 1.9 µm for the outer bark and 13.8 ± 2.0 µm for the inner bark (*n* = 10 each; compared via t-test, *p* = 0.118).

### 2.2. Dynamic Drop-Weight Tests

**Figure 5:**
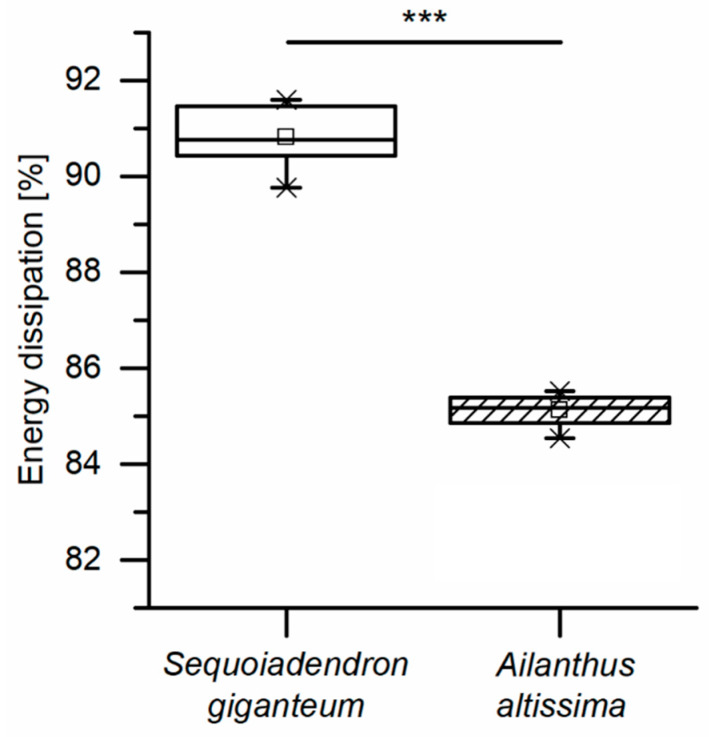
Energy dissipation of bark samples of *S. giganteum* and *A. altissima* during dynamic drop-weight tests (statistical test: t-test; *n* = 10 for *S. giganteum*, *n* = 8 for *A. altissima*). Significant differences are marked by asterisks, with the levels of significance indicated as follows: *** *p* < 0.001.

The energy dissipation capability of the bark of *S. giganteum* amounts to 90.8% (Figure 5), meaning that 90.8% of the energy introduced by the impactor is dissipated by the tree bark. In comparison, the energy dissipation capability of *Ailanthus altissima*, a tree species that is never threatened by rockfall in its natural habitat, is 85.2%, which is significantly lower than that of *S. giganteum*. High energy dissipation capabilities of about 90% are also found in other impact-protecting plant structures. e.g., the peel of the pomelo fruit (*Citrus grandis*) [29]. This proves the suitability of the bark of *S. giganteum* as an effective impact protection and shows that *S. giganteum* bark is significantly more capable of protecting the underlying vital tissues, such as the cambium, during an impact event. Converting the dissipated energy into an energy density yields a mean value of 0.139 ± 0.016 MJ/m^2^ when taking into account the whole volume of each tested sample, i.e., related to the quadratic basal area of the sample. When considering only the compressed volume (i.e., the bark volume directly under the impactor), the energy density increases to 0.157 ± 0.010 MJ/m^2^ (see Appendix A for further details on these two approaches).

**Figure 6:**
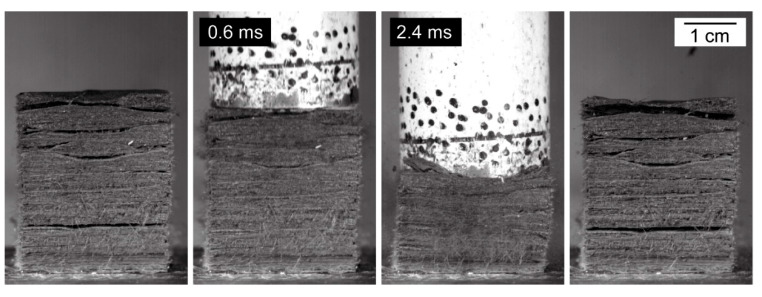
Images extracted from a high-speed video of a dynamic drop-weight test of *S. giganteum* bark, revealing its nonuniform compaction behavior. From left to right: bark sample before impact, bark sample 0.6 ms and 2.4 ms after first contact with the impactor, bark sample after impact test.

Images extracted from a high-speed video of a typical drop-weight test of *S. giganteum* bark (Figure 6) indicate a nonuniform compaction behavior of the outer bark. In the first phase, mainly the air-filled spaces between the fiber layers are compressed. Only in the second phase, the layers themselves are compressed. Thereby—most likely—both the network of fibers and the fibers themselves are compressed. Furthermore, no considerable transversal expansion of the samples can be observed during compression. It is also noteworthy that, after an impact event, the bark macroscopically recovers its original shape to a large extent (however, not entirely). This delayed recovery indicates a viscoelastic component of the bark deformation behavior, as will be characterized in more detail later on, in the quasistatic cyclic compression tests.

### 2.3. Quasistatic Compression Tests

#### 2.3.1. Quasistatic Compression Tests (One Loading Stage)

**Figure 7:**
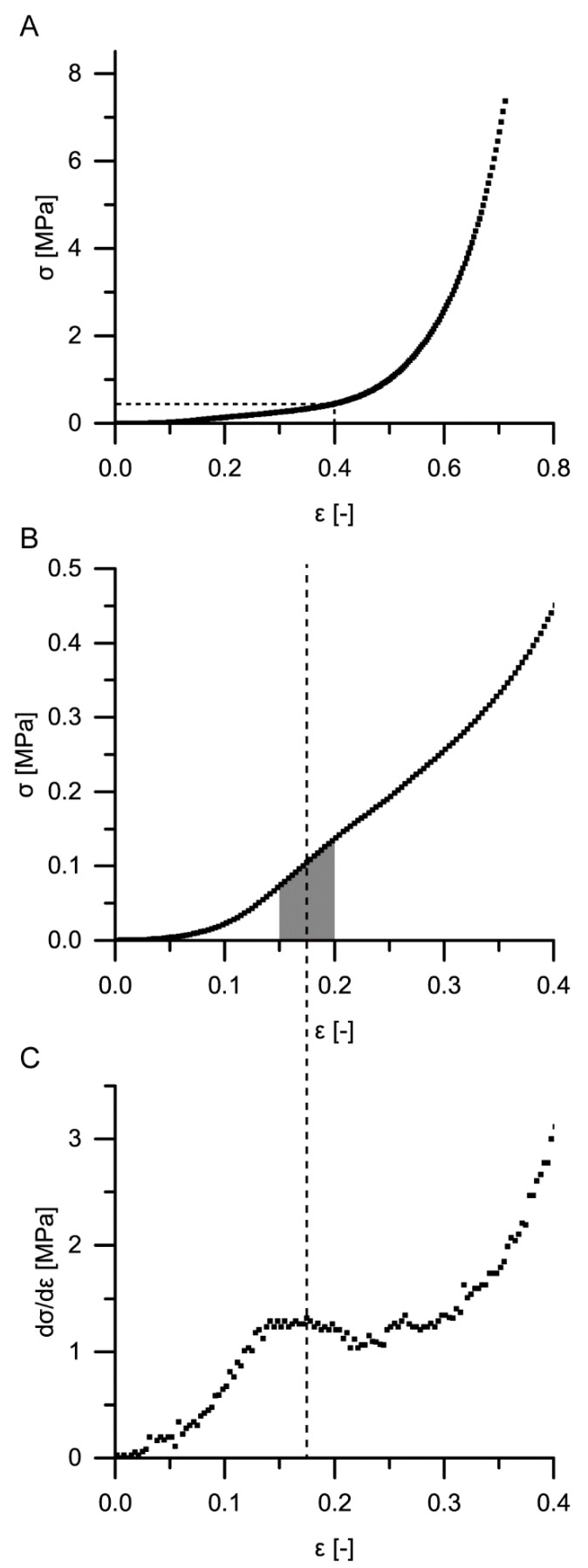
Exemplary stress–strain curves (**A**,**B**) and slope–strain curve (**C**) of quasistatic compression tests (one loading stage) of *S. giganteum* bark samples (strain rate 0.1 min^−1^). (**A**) Whole stress–strain curve. (**B**) Enlarged detail of this stress strain curve, until 40% strain. The grey area marks one of the two methods to derive a structural Young’s modulus from the stress–strain curves (slope of the best-fit line between 15% and 20% strain). The dashed lines in (**B**) and (**C**) refer to the second method, namely the strain at which the maximum slope until 20% strain is observed in (**C**), where the slope of the stress strain curve from (**B**) is plotted against the strain, allowing to extract the maximum slope until 20% strain.

**Figure 8:**
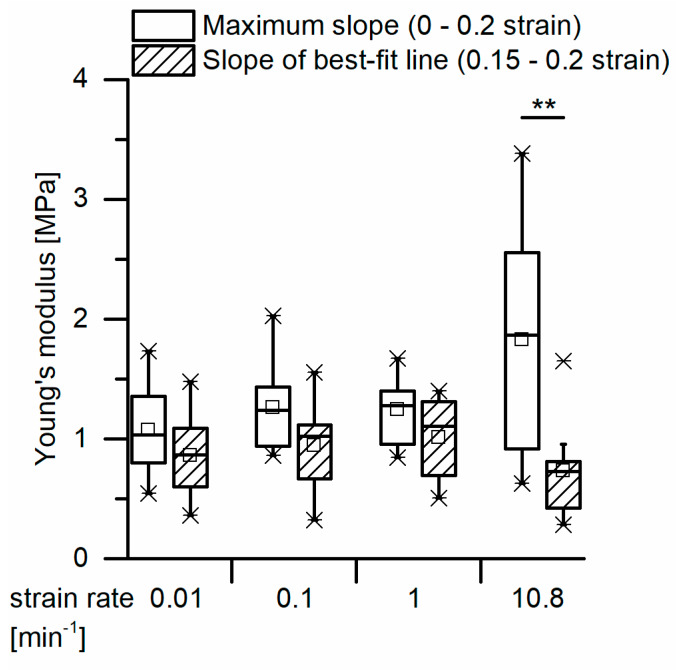
Comparison of the quasistatic compressive structural Young’s moduli derived by the two different approaches, at several strain rates. In one approach, the Young’s modulus is calculated as the maximum slope of the stress–strain curve until 20% strain. In a second approach, it is calculated as the slope of the best-fit line between 15% and 20% strain (statistical tests: Kruskal–Wallis test to compare the first mentioned method, ANOVA to compare the second mentioned method, Mann–Whitney U-tests to test the pairs for each strain rate; *n* = 10 for each group). Significant differences are marked by asterisks, with the levels of significance indicated as follows: ** *p* < 0.01.

Quasistatic compression tests on the *S. giganteum* bark including merely one loading stage reveal steadily rising stress–strain curves (see Figure 7A,B for exemplary stress–strain curves). In general, the stress–strain curves resemble those of typical elastomeric foams (compare, e.g., [18]). However, a distinct plateau region is absent; thus, a clear distinction of several phases during compression (especially a distinction between a linear elastic and a plateau regime) cannot be observed. Therefore, in order to determine a reproducible structural Young’s modulus for each sample, two different approaches were chosen (Figure 7B,C): (1) the slope of the best-fit line between 15% and 20% strain was defined as the Young’s modulus; (2) the maximum slope until a strain of 20% was calculated and defined as Young’s modulus. This second approach follows a procedure suggested by [30]. Although structural Young’s moduli calculated by using the maximum slope until 20% strain were always larger than those calculated via the best-fit line between 15% and 20%, no significant differences could be found when comparing the two approaches statistically, except for a strain rate of 10.8 min^−1^ (Figure 8), for which the maximum slope methods give a significantly higher value. However, the high strain rate of 10.8 min^−1^ might already overrate time-dependent (i.e., viscoelastic) properties of the bark and might therefore not be representative for quasistatic compression tests. For the other strain rates (i.e., from 0.01 to 1 min^−1^), the Young’s moduli do not vary significantly for any of the two evaluation approaches. For these strain rates, their mean Young’s moduli amount between 0.87 and 1.11 MPa for the first approach and between 1.03 and 1.28 MPa for the second approach.

#### 2.3.2. Quasistatic Cyclic Compression Tests

Both in the case of cyclic test with stepwise increasing maximum strain and cyclic test with unchanged maximum strain, a time-dependent (i.e., viscoelastic) behavior when loading and unloading the bark can be demonstrated. During the unloading cycles, the bark deformation is partly recovered, but not completely (i.e., the strain decreases during the unloading cycle, but when the stress reaches 0 MPa, the strain does not fully go back to zero and a residual strain remains). The magnitude of the residual strain depends on the strain rate.

##### Cyclic Test with Stepwise Increasing Maximum Strain

**Figure 9:**
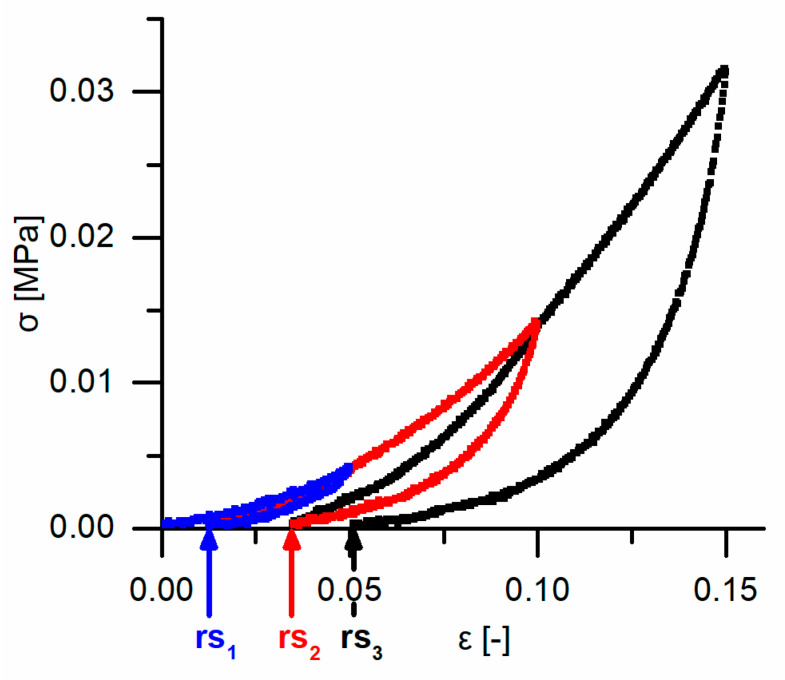
Exemplary stress–strain curve of a quasistatic stepwise cyclic compression test (strain rate 1 min^−1^) consisting of three cycles, as sketched in Figure 16. The stress–strain curve of the first cycle is colored in blue, that of the second cycle is in red and that of the third cycle is in black. The residual strains of the first, second and third cycles are marked with rs_1_, rs_2_ and rs_3_, respectively.

**Figure 10:**
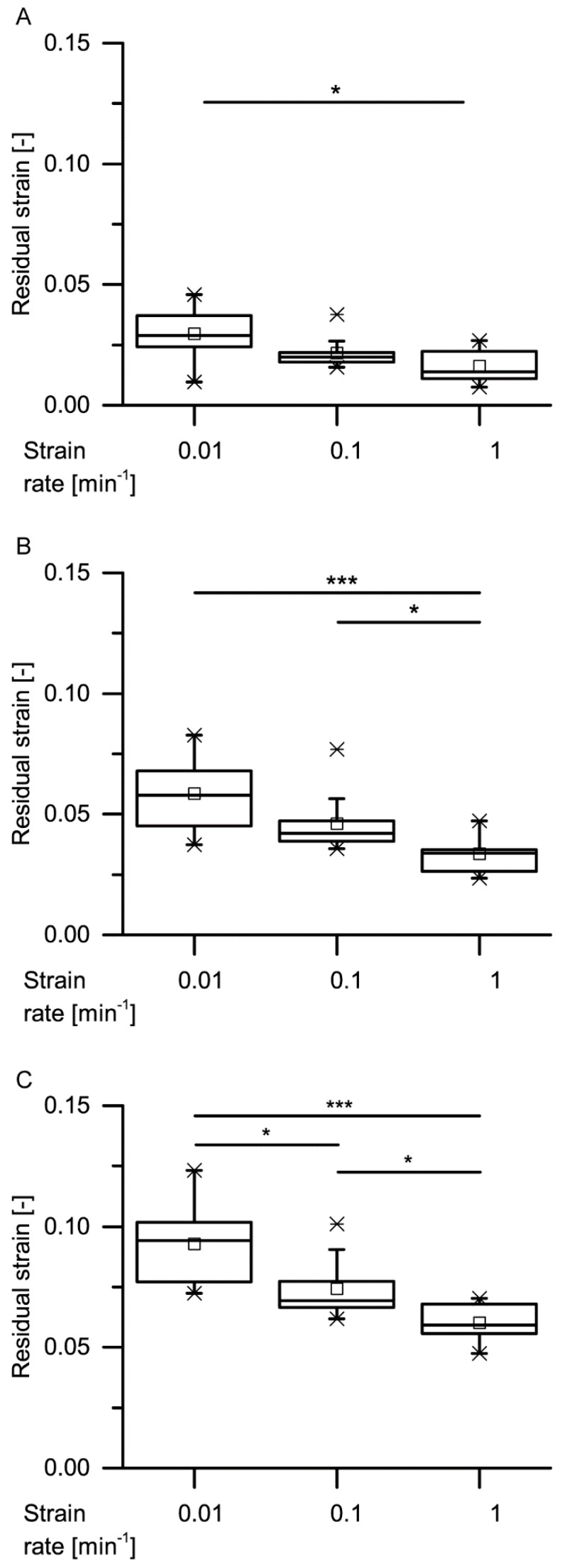
Residual strains of the first (**A**), second (**B**) and third (**C**) cycles of the quasistatic stepwise cyclic compressions tests, at several strain rates (statistical test: Kruskal–Wallis test; ***n*** = 10 for each group). Significant differences are marked by asterisks, with the levels of significance indicated as follows: * *p* < 0.05, *** *p* < 0.001.

An exemplary stress–strain curve of a cyclic test with stepwise increasing maximum strain (Figure 9) shows the time-dependent (viscoelastic) behavior. During each of the three cycles (i.e., stepwise until 15%), the unloading curve differs markedly from the loading curve, proving that the bark is not characterized by merely elastic deformation. The shape of the bark samples gets restored to a large extent upon unloading (i.e., the strain markedly decreases during the unloading cycle), and stress–strain hysteresis loops can be observed between the loading and unloading cycles, proving the presence of a viscoelastic deformation behavior. The residual strain increases from cycle to cycle and decreases with increasing strain rate (Figure 10). While only the highest and the lowest strain rates differ significantly during the first cycle, the intermediate and the highest strain rates also differ significantly during the second cycle, and all strain rates differ significantly during the third cycle. These strain rate dependencies further support the presence of a viscoelastic deformation behavior.

##### Cyclic Test with Unchanged Maximum Strain

**Figure 11:**
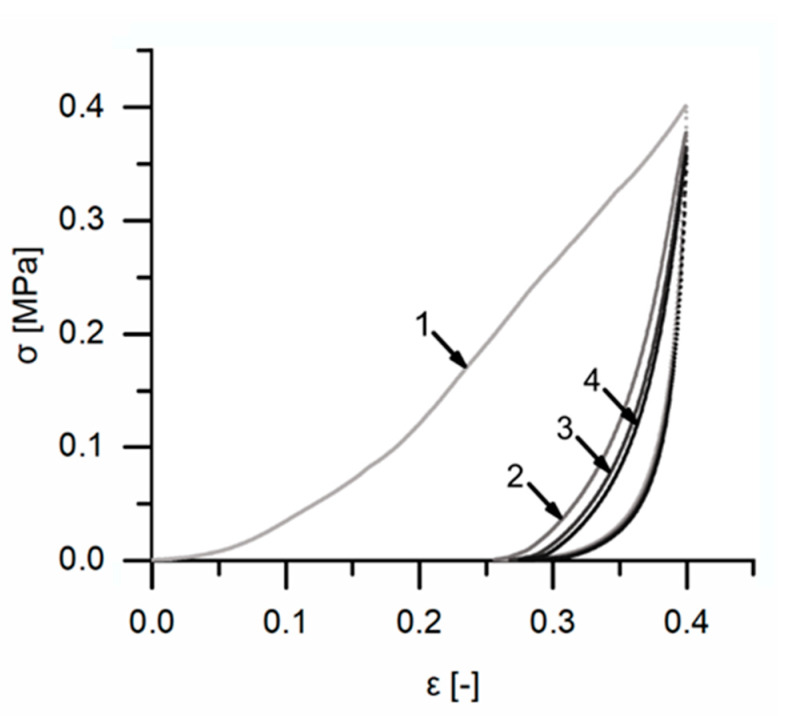
Exemplary stress–strain curve of a quasistatic cyclic compression test with unchanged maximum compression (strain rate 1 min^−1^) consisting of four cycles, as sketched in Figure 16. The loading curve of each cycle is marked with an arrow and labeled according to the number of the corresponding cycle. The unloading curves are superimposed.

**Figure 12:**
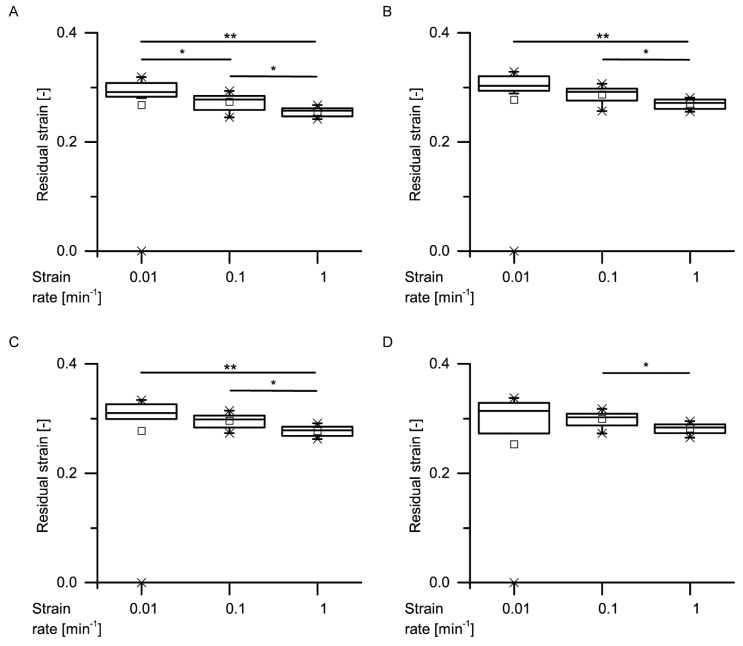
Residual strains of the first (**A**), second (**B**), third (**C**) and fourth (**D**) cycles of the quasistatic cyclic compression tests with unchanged maximum compression, at several strain rates (statistical test: Kruskal–Wallis test; *n* = 10 for each group). Significant differences are marked by asterisks, with the levels of significance indicated as follows: * *p* < 0.05, ** *p* < 0.01.

An exemplary stress–strain curve of a cyclic test with unchanged maximum strain (Figure 11) also supports the insights already gained during the cyclic test with stepwise increasing maximum strain, for higher loading scenarios (up to 40% strain). The unloading curve of each cycle differs from the loading curve, and the strain markedly decreases during the unloading cycle instead of remaining constant (i.e., the deformation behavior is neither purely elastic nor plastic). Comparable to the cyclic tests involving a stepwise increasing maximum compression, a strain rate dependency can be demonstrated for all four cycles, with a significantly decreasing residual strain with increasing strain rate (Figure 12).

**Figure 13:**
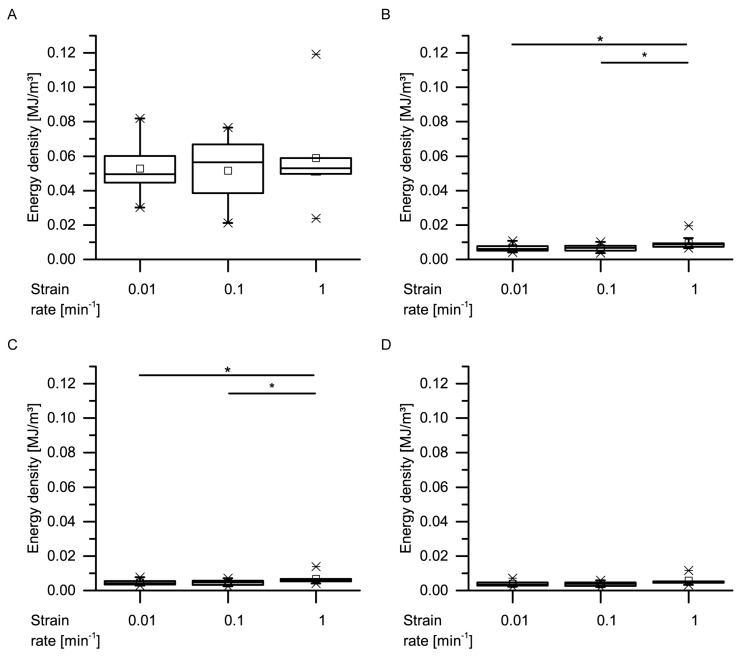
Energy densities of one whole cycle (dissipated energy E_d_) of the quasistatic cyclic compression tests with unchanged maximum compression, for the first (**A**), second (**B**), third (**C**) and fourth (**D**) cycles (statistical test: Kruskal–Wallis test; *n* = 10 for each group). Significant differences are marked by asterisks, with the levels of significance indicated as follows: * *p* < 0.05.

**Figure A1:**
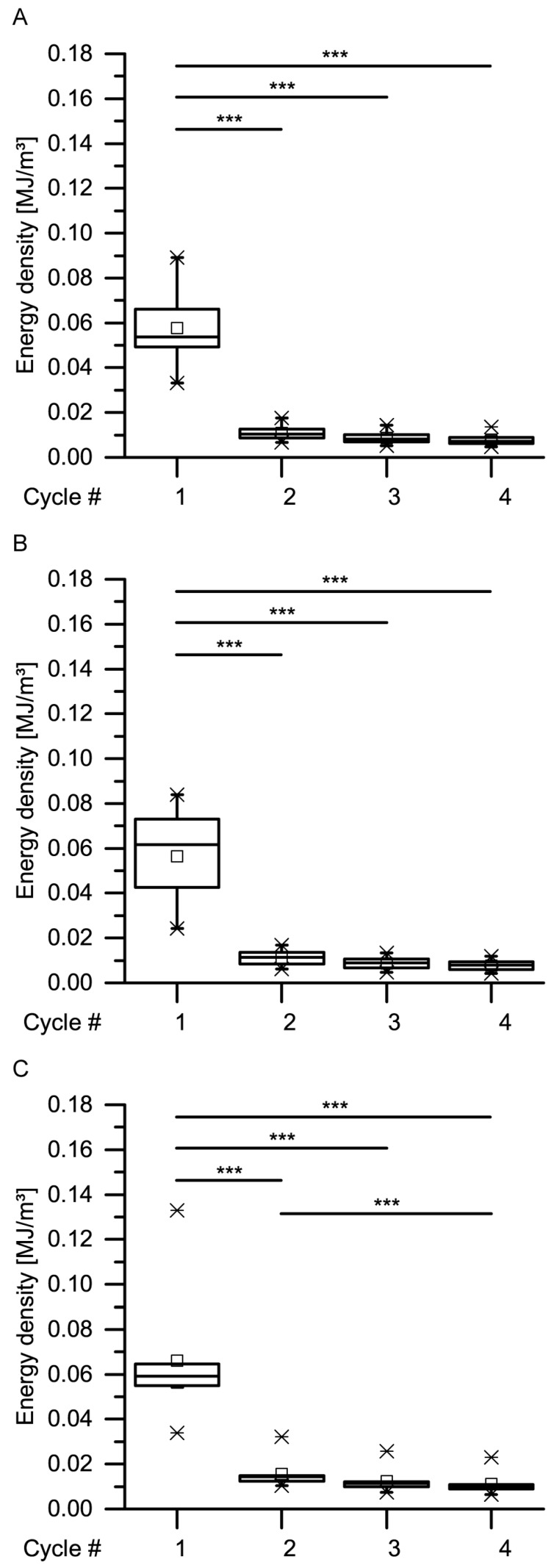
Energy densities of the loading curves (absorbed energy E_a_) of the quasistatic cyclic compression tests with unchanged maximum compression, at strain rates of 0.01 min^−1^ (**A**), 0.1 min^−1^ (**B**) and 1 min^−1^ (**C**) (statistical test: Kruskal–Wallis test; *n* = 10 for each group). Significant differences are marked by asterisks, with the levels of significance indicated as follows: *** *p* < 0.001.

**Figure A2:**
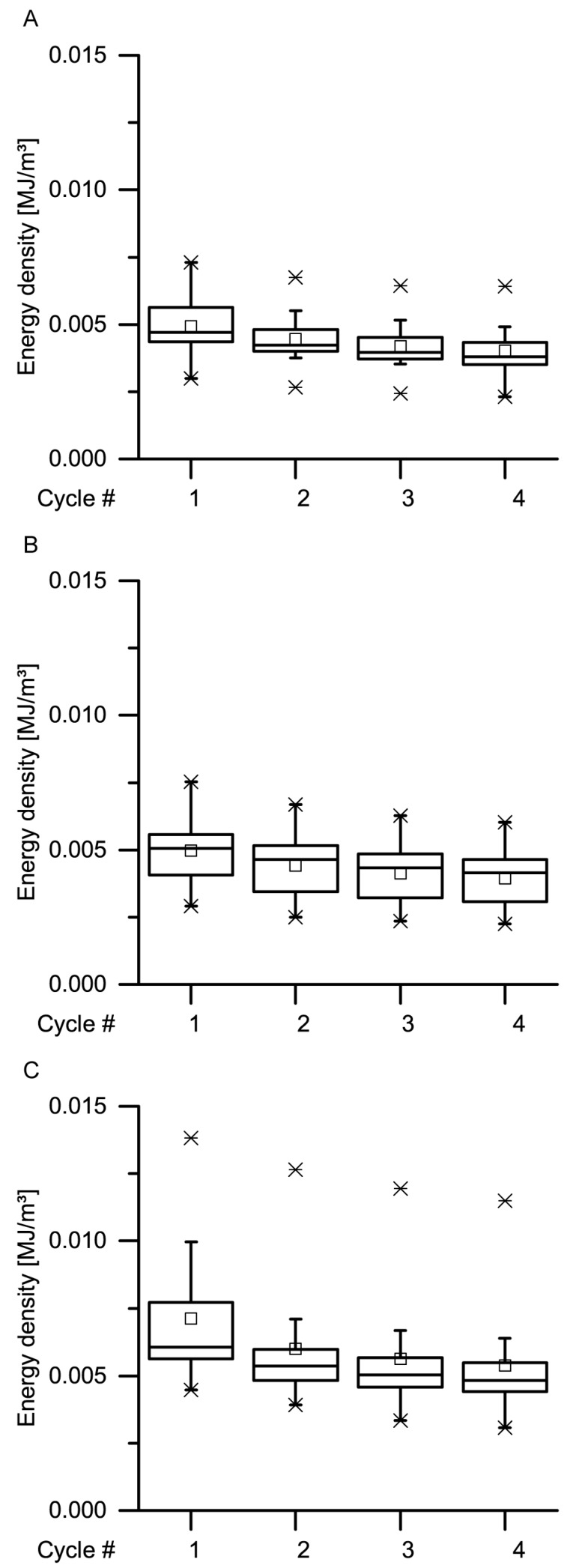
Energy densities of the unloading curves (released energy E_r_) of the quasistatic cyclic compression tests with unchanged maximum compression, at strain rates of 0.01 min^−1^ (**A**), 0.1 min^−1^ (**B**) and 1 min^−1^ (**C**) (statistical test: Kruskal–Wallis test; *n* = 10 for each group).

A comparison of the energy densities shows that the median values of the absorbed energy densities E_a_ range from 0.0072 MJ/m^2^ (cycle 4 at strain rate 0.01 min^−1^) to 0.0616 MJ/m^2^ (cycle 1 at strain rate 0.1 min^−1^) (Figure A1). The energy density of the loading curve of the first cycle is always significantly higher than that of the loading curve of the second, third and fourth cycles. No other significant differences could be found except at the highest strain rate (1.0 min^−1^), where the energy density is significantly higher for the loading curve of the second cycle compared to the fourth cycle. Concerning the unloading curves, the median released energy densities E_r_ range from 0.0038 MJ/m^2^ (cycle 4 at strain rate 0.01 min^−1^) to 0.006 MJ/m^2^ (cycle 1 at strain rate 1 min^−1^) (Figure A2). However, no significant differences between the cycles were found at any strain rate. In order to directly test the influence of the strain rate on the energy density of each cycle, the energy densities of the loading and unloading curves were not regarded separately as before, but combined (i.e., the area under the unloading curve was subtracted from the area under the loading curve, yielding the dissipated energy density E_d_ of each cycle). This approach allows the evaluation of the influence of the strain rate on the overall energy dissipation of the bark. The mean values range from 0.0034 MJ/m^2^ (cycle 4 at strain rate 0.01 min^−1^) to 0.0565 MJ/m^2^ (cycle 1 at strain rate 0.1 min^−1^), and only the energy densities of the strain rate of 1 min^−1^ are significantly higher than for the other strain rates for the second and third cycles (Figure 13).

In addition to comparing the influence of cycle number and strain rate on energy absorption or energy dissipation, the energy densities dissipated during the first cycles of the quasistatic cyclic compression tests with unchanged maximum strain can be compared to the energy densities dissipated during the dynamic drop-weight tests (cf. Section 2.2). Thereby, the energy densities of the dynamic drop-weight tests are between 2.5 and 2.8 times larger when considering the whole bark volume during the drop-weight tests, and between 2.8 and 3.2 times larger when considering only the compressed bark volume during the drop-weight tests. However, when comparing these values, it needs to be considered that the testing velocities and strain rates are much higher during the dynamic drop-weight tests (12,238 ± 857 min^−1^ for the maximum strain rate and 7522 ± 602 min^−1^ for the mean strain rate during the loading phase) compared to the quasistatic tests (between 0.01 and 1 min^−1^) (see Appendix D).

### 2.4. Microtensile Tests

**Figure 14:**
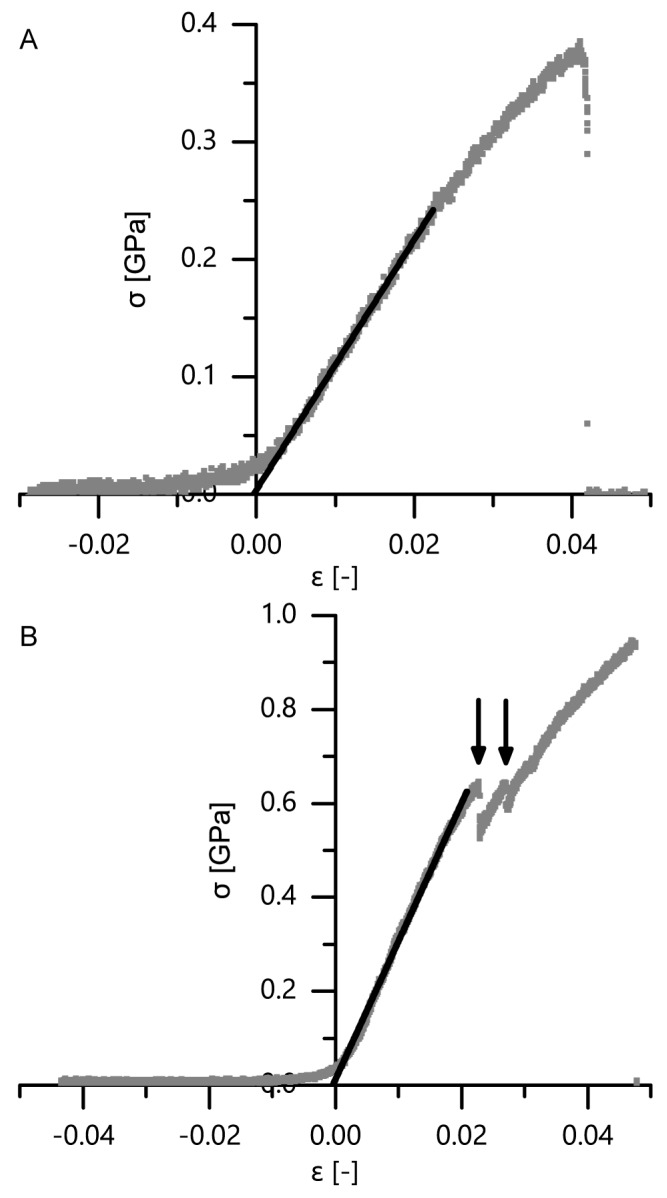
Exemplary stress–strain curves of microtensile tests on a single extracted single bark fiber of the outer bark (**A**) and an extracted pair of interconnected bark fibers of the outer bark (**B**). Whereas typically no prefailure events were observed during tensile tests on single bark fibers, such prefailure events were abundant during tensile tests of fiber pairs (marked with arrows). The black lines in the graphs indicate the slope of the best-fit line of the initial linear range used to determine zero strain.

**Figure 15:**
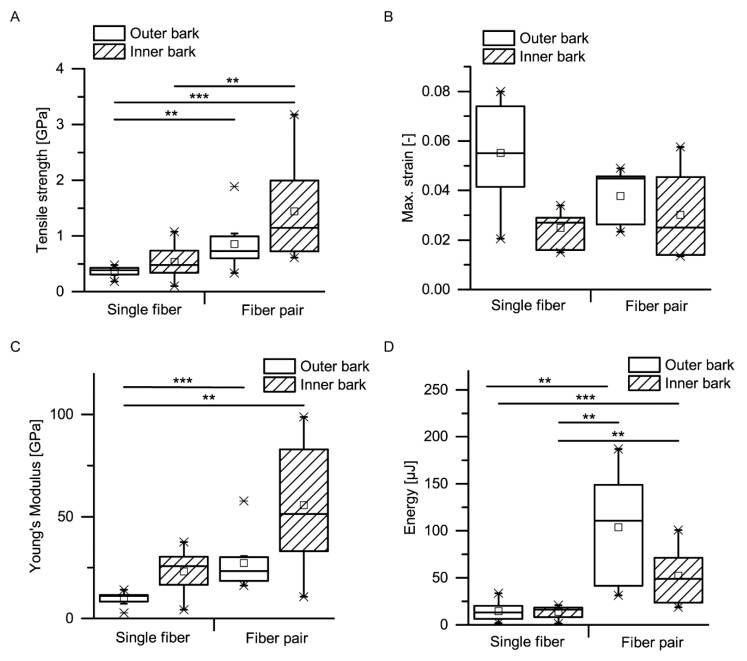
Tensile strength (**A**), maximum strain (**B**), Young’s modulus (**C**) and dissipated energy (**D**) of microtensile tests, comparing both single fibers vs. fiber pairs and samples extracted from the outer bark vs. samples extracted from the inner bark (statistical test: Kruskal–Wallis test; *n* = 10 for TS/sf/ib and YM/sf/ib; *n* = 9 for TS/ob/sf and YM/ob/sf; *n* = 8 for TS/fp/ob, TS/fp/ib, MS/sf/ob, YM/fp/ob, YM/fp/ib and E/sf/ob; *n* = 7 for MS/sf/ib and E/sf/ib; *n* = 6 for MS/fp/ib and E/fp/ib; *n* = 5 for MS/fp/ob and E/fp/ob). TS = tensile stress, MS = max. strain, YM = Young’s modulus, E = energy, sf = single fiber, fp = fiber pair, ob = outer bark, ib = inner bark. Significant differences are marked by asterisks, with the levels of significance indicated as follows: ** *p* < 0.01, *** *p* < 0.001.

Exemplary stress–strain curves of microtensile tests of single bark fibers and fiber pairs are displayed in Figure 14. Typically, no prefailure events occurred when single fibers were tested, whereas such prefailure events were typical for fiber pairs. The latter can be related to the finding that in fiber pairs the fibers are not connected continuously along the fiber length (Figure 4). Instead, several zones occur in which the two fibers are not visibly connected to each other, resulting in several predetermined weak spots per fiber pair, probably causing the observed prefailure events. However, despite this seemingly loose interconnection between two adjacent fibers, several mechanical properties (i.e., the tensile strength, Young’s modulus and dissipated energy) are significantly higher in fiber pairs than in single fibers (Figure 15A,C,D): the median tensile strength (± confidence interval) amounts 0.39 ± 0.06 GPa for single fibers in the outer bark, 0.73 ± 0.19 GPa for fiber pairs in the outer bark, 0.48 ± 0.19 GPa for single fibers in the inner bark and 1.14 ± 0.61 GPa for fiber pairs in the inner bark; the median Young’s modulus amounts 11.1 ± 1.7 GPa for single fibers in the outer bark, 23.2 ± 6.2 GPa for fiber pairs in the outer bark, 25.6 ± 5.5 GPa for single fibers in the inner bark and 51.4 ± 25.3 GPa for fiber pairs in the inner bark; the median dissipated energy amounts 13.2 ± 6.6 µJ for single fibers in the outer bark, 111.1 ± 75.6 µJ for fiber pairs in the outer bark, 16.1 ± 4.1 µJ for single fibers in the inner bark and 49.0 ± 29.7 µJ for fiber pairs in the inner bark. This is remarkable, as the tensile strength and Young’s modulus take into account the increased cross-section when testing two fibers instead of one. However, while combining fibers to form fiber pairs results in improved mechanical properties, the extensibility of fiber pairs remains unaffected, i.e., the maximum strain of fiber pairs does not decrease significantly compared to single bark fibers (Figure 15B).

Apart from comparing single fibers to fiber pairs, a comparison between the outer and the inner bark becomes relevant when attempting to explain the giant sequoia bark’s high energy dissipation capability compared to other bark, such as that of *A. altissima* (Figure 5). No significant differences can be found for the tensile strength, the maximum strain, the Young’s modulus or the dissipated energy when comparing single fibers and fiber pairs from outer bark and inner bark (Figure 15A–D). Thus, a potential degradation, wear or abrasion of fibers in the outer bark seems to have no significant negative (weakening) effects on their mechanical properties. However, even if differences are not significant, the general trend goes towards higher tensile strengths and Young’s moduli for fibers and fiber pairs in the inner bark, whereas the dissipated energy is highest for fiber pairs of the outer bark (even when taking into account that the cross-sectional area of fiber pairs is about twice that of single fibers). Furthermore, the extensibility of fibers and fiber pairs tends to be higher in the outer bark than in the inner bark.

## 3. Discussion

For a better understanding of the very specific composition and compaction behavior of the bark of the giant sequoia, the most relevant aspects will be compared to other selected biological and technical structures and materials. Concerning the stress–strain curves during quasistatic compression tests, the bark rather resembles an elastomeric open-pore foam, although the characteristic compression regimes of a typical elastomeric open-pore foam cannot clearly be distinguished for the bark. Instead, a smooth transition between the regimes can be observed, and no distinct plateau region can be found. During the high-speed videos of the dynamic drop-weight tests, macroscopically, a nonuniform compaction behavior can be observed. This also holds true for other biological structures, such as the pomelo peel [21,22], where the nonuniform compaction behavior is mainly attributed to the presence of lignified and branched vascular bundles, which can be regarded as fiber network in an open-pore parenchymatous foam layer. However, the bark of the giant sequoia (mainly) consists of fibers without any matrix in between; thus, its nonuniform compaction behavior can be mainly attributed to its layered structure on two orders. In contrast to these two biological structures, most technical elastomeric foams show a uniform compaction behavior. Concerning the absence of a transversal expansion during impact or compression (Figure 6), the bark of the giant sequoia again can be compared to many polymer foams, which might even exhibit a negative Poisson’s ratio [31,32,33]. From a structural point of view, the bark resembles a foam, especially when compared to the barks of most other tree species. It consists of a high amount of fibers and a considerable proportion of air-filled space, and the fibers form a network due to the interwoven fiber layers (Figure 2 and Figure 3). Thereby, the bark can be described as an open-pore foam-like structure with the bark fibers representing the ‘foam struts’. A similar comparison has also been made for the pomelo peel [21,22], where a considerable proportion of the peel’s impact-damping capabilities and viscoelastic behavior was attributed to the liquid cell-sap in the living parenchymatous cells, which represent the ‘foam struts’ of the peel. In contrast, in the giant sequoia bark, the thick cell walls of the dead fibers occupy almost the whole cell volume, leaving no or almost no cell lumen. Thus—besides water chemically bound in the cell wall—only small quantities of “free” water can be stored in this cell lumen, which is even further reduced upon drying of the bark prior to testing. Therefore, only a minor influence of liquid stored within the fibers on the deformation behavior is to be expected for the giant sequoia bark. The viscoelastic component occurring during bark compression seems to be a material and/or structure immanent property rather than being based on liquid displacement or squeezing.

Concerning wood and wood composites, the authors of [34] stated that during constant loading, initially, all the deformation is elastic. With elapsing time, the viscous (i.e., nonelastic) component responds, resulting in a decrease of the elastic component. In general, this holds true also for the bark samples under both quasistatic stepwise cyclic compression and cyclic compression with unchanged maximum compression. Even though the residual strains (representing the nonelastic components) significantly decrease with increasing strain rate for almost all cycles, a considerable amount of nonelastic components is still present during all these tests (Figure 9, Figure 10, Figure 11 and Figure 12).

When comparing the energy dissipation mechanisms of the giant sequoia bark with other biological protection structures such as pomelo peel [21,22], macadamia seed shell [25,26] and the coconut fruit wall [17,28], it could be shown that all these structures protect the inlying organs or tissues during impact events mainly by the reduction of force peaks. Hard and tough structures such as the coconut endocarp and macadamia seed shell dissipate energy by crack path deviation and elongation—energy dissipation mechanisms which are not present in the pomelo peel or giant sequoia bark, whose energy dissipation mechanisms are based on pronounced viscoelastic deformation.

With regard to the energy densities dissipated during both dynamic drop-weight tests and quasistatic cyclic compression test, it is worthwhile to consider whether the dissipated energy density of the giant sequoia bark depends on the strain rate or not. When considering only the (most relevant) first cycle of the quasistatic cyclic compression tests with unchanged maximum compression, no strain rate dependency can be observed (Figure 13). However, as these strain rates only range from 0.01 to 1 min^−1^, they do not cover a very wide range. When thus comparing the dissipated energy density between the slowest quasistatic compression tests (a very slow strain rate of 0.01 min^−1^) and the dynamic drop-weight tests (a comparably high strain rate of 7.5 × 10^3^ min^−1^), an increase of about 3.2 times is found. The dissipated energy density thus markedly depends on the strain rate, if a possible influence of the different methods is not taken into consideration. In general, this is not surprising, as many materials and structures exhibit a strain rate dependency of their mechanical properties. For example, the authors of [35] examined the strain rate dependency of several mechanical properties of several general-purpose plastics, namely acrylonitrile butadiene styrene (ABS), high-density polyethylene (HDPE), polypropylene (PP) and polyvinylchloride (PVC). The loading scenarios of their compression experiments (the strain rate of the slowest quasistatic tests amounted 7.2 × 10^−2^ min^−1^, while the strain rate of the dynamic split Hopkins tests amounted between about 3.6 × 10^4^ min^−1^ and 4.7 × 10^4^ min^−1^) were approximately comparable to those of the present study. When comparing the dissipated energy densities between the quasistatic and the dynamic tests, they found an increase of about 1.19 times for PVC, 1.34 times for HDPE, 1.49 times for ABS and 2.47 times for PP. However, the authors of [36] stated a low strain-rate dependency of biocomposites compared to technical composites and referred this difference, among others, to the natural fibers present in the biocomposites, a finding that would also indicate a low strain-rate dependency for the fibrous giant sequoia bark. However, the strain rate dependency of the giant sequoia bark is, with an increase of a factor of up to 3.2, considerably higher than for the tested plastics. This high strain-rate dependency for the dissipated energy densities of the giant sequoia bark becomes relevant during rockfall events, representing high-velocity impacts. The bark of the giant sequoia can be considered as adapted to the naturally occurring conditions during high-energy, high-velocity impact events.

For a calculation of the structural Young’s modulus under compression for the bark samples, it is essential to identify the boundaries of the linear elastic region. However, in the case of the quasistatic compression tests, the identification of the linear elastic region is not straightforward. For cellular solids (e.g., an elastomeric foam), the linear elastic region is often referred to as occurring until ca. 0.05 strain (e.g., [18,19]), or the boundaries of this region are calculated involving physical quantities such as the density of the cellular material and/or the density of the cell wall material [18]. However, in the case of the giant sequoia bark, calculating the density of the cellular material is difficult, as several cell types and layers are involved, and a mean density of the cell wall material cannot simply be calculated or assumed. Furthermore, the aforementioned assumed boundary of 0.05 strain seems to not apply with regard to the stress–strain curves found for the giant sequoia bark (Figure 7). Thus, different approaches were used for calculating the Young’s modulus. The Young’s moduli of the two selected methods (i.e., calculation of the maximum slope between 0 and 0.2 strain as suggested by [30] and the slope of the best-fit line between 0.15 and 0.2 strain) do not differ significantly for the strain rates between 0.01 and 1 min^−1^. Amounting between 0.87 and 1.28 MPa, they are low when comparing them to values found for, e.g., technical foams or other biological “foam-like” materials such as cork. When considering the density of the giant sequoia bark (about 0.23 g/cm^2^, as measured by [13]), the abovementioned materials of comparable density are typically characterized by a Young’s modulus of 10 MPa or more [37], being at least ca. 8 times higher than those measured for the giant sequoia bark. The high energy dissipation capability of the giant sequoia bark is remarkable, despite its comparatively low Young’s modulus. However, this goes along with the high energy dissipation capability found in the microtensile tests on single bark fibers and fiber pairs.

A comparison of mechanical properties of single fibers and fiber pairs as determined in microtensile tests proves that combining single fibers to fiber pairs does not worsen their extensibility (no significant differences in maximum strain) but significantly improves the other mechanical properties, such as tensile strength, Young’s modulus and dissipated energy (Figure 14 and Figure 15). When transferring this mechanical behavior to the whole bark structure, it most probably results in an advantageous response during and after an impact. During an impact, the bark is capable of protecting the cambium due to its beneficial mechanical properties. After an impact, the relaxation of the compressed bark towards its original, nondeformed, configuration is facilitated due to the high extensibility of the connected and unconnected fibers (especially in the thick outer bark), which is beneficial for a quick recovery of the protective functions of the bark after an impact.

The development of mechanical properties of fibers during the ontogenesis of the bark favors the bark’s protective potential during impact events. Although no significant differences can be found between outer and inner bark concerning tensile strength, maximum strain, Young’s modulus or dissipated energy of single fibers and fiber pairs, a general trend goes towards higher tensile strengths and Young’s moduli for fibers and fiber pairs in the inner bark, whereas the dissipated energy is highest for fiber pairs of the outer bark. Only the inner bark contains living cells, whereas all cells in the outer bark are dead and thus not metabolically active [1]. As a consequence, the entire dead outer bark cannot be physiologically regenerated after injury or other forms of degradation, which goes along with the general trend of slightly worsening most mechanical properties in the outer bark, with exception of the dissipated energy being highest for fiber pairs in the outer bark. This means that the protective role of the outer bark can at least be preserved, if not tendentially improved, compared to the inner bark. The finding that the dimensions of the fiber cross-section do not differ between dead outer and living inner bark indicates that all examined fibers were already fully mature. Furthermore, it can be concluded that the fibers in the outer bark are not visibly altered despite being exposed to diverse environmental and growth-related influences during the naturally occurring lateral expansion of the bark. However, the authors did not examine the composition of the cell wall, where ageing processes might bring about an effect. Thus, besides the above-mentioned mechanical wear or abrasion, potential differences in the cell wall composition between outer and inner bark are most likely the cause for the differing mechanical properties of fibers between the outer and inner bark (Figure 15).

The mechanical properties of single bark fibers and fiber pairs are in the range of other plant and technical fibers and fiber bundles tested in tension (Table 1). The tensile strength of single giant sequoia fibers from the inner and outer bark are in the middle region compared to other single plant or technical fibers, whereas the tensile strength of fiber pairs is in the upper region compared to bundles of natural fibers. The maximum strain of single fibers and fiber pairs falls in the upper region compared to other natural plant fibers, except for single cotton fibers that can reach much higher maximum strains. The Young’s modulus of single fibers of the inner bark falls in the middle region, while that of single fibers of the outer bark fibers falls in the lower region. The Young’s modulus of fiber pairs of the inner bark falls in the upper region, while that of fiber pairs of the outer bark falls in the middle region. The energy dissipation capacity of the fibers of the outer bark is at least equal to the inner bark, despite Young’s modulus and tensile strength being smaller (Figure 15); literature values for comparison of energy dissipation with other plant fibers were not found. Furthermore, only single fibers and fiber pairs were considered in this comparison to other plant and technical fibers; thus, the energy dissipation will most likely stand out even more when considering the whole bark as a highly sophisticated layered structure.

As already mentioned in the introduction, an implementation of several hierarchical levels of a biological role model has already been proven beneficial for the optimization of energy dissipation of technical products other than concrete- and ceramic-based components [24,25]. The findings of the present paper are relevant for the application in bio-inspired fibrous concrete and ceramic components for building construction [16,27]). However, a potential application is not limited to concrete or ceramic components but includes all classes of fiber-reinforced materials. Hereby, the structuring of the giant sequoia bark on several hierarchical levels including the high cell wall content of the bark fibers, the almost rectangular fiber cross-section, the mode of connection between adjacent bark fibers and the arrangement of fibers in two orders of partially interwoven layers serve as potential inspirations. A transfer into concrete- and ceramic-based components will potentially lead to an increased impact resistance and energy dissipation behavior of the bio-inspired structures and might help to increase earthquake security by an improved energy dissipation and a benign failure behavior.

## 4. Materials and Methods

### 4.1. Plant Material

Two giant sequoia (*Sequoiadendron giganteum* Lindl.) trees and one tree of heaven (*Ailanthus altissima* (Mill.) Swingle) were felled, and bark samples were used for the various analysis and testing methods. After felling of the trees, stem segments including both intact bark (defined here as comprising all tissues outside the vascular cambium) and xylem were stored at room temperature and a relative humidity of typically about 60% until further processing for the experiments. One *S. giganteum* tree (samples thereof were used for the dynamic drop-weight tests and the stained thin sections, see below) was about 90 years old when felled at the Botanic Garden Freiburg, the second *S. giganteum* tree (samples thereof were used for µCT, SEM, quasistatic compression tests and microtensile tests) was about 32 years old when felled in the city area of Freiburg i. Br., Germany. The *A. altissima* tree was about 70 years old when felled at the Botanic Garden Freiburg. All samples used in the studies presented here were taken approximately from breast height, except the samples used for the dynamic drop-weight tests (see below) that were taken from heights of 17.9 m (*A. altissima*) and 19 m (*S. giganteum*) above ground. The sampling height for the dynamic drop-weight tests deviates from that of all other studies because approximately matching sampling heights are required for a reasonable comparison between the two tree species, which was only the case for the sampling heights of 17.9 and 19 m.

### 4.2. µCT Scans, SEM and Thin Sections

Samples of the whole bark of *S. giganteum* were used for the µCT scans. µCT scans were performed using a Skyscan 1272, Bruker microCT, Kontich, Belgium. The µCT data were processed and volume-rendered (in the following, the term ‘segmented’ will be used) using the CT-Analyzer (V. 1.16.4.1, Bruker microCT, Kontich, Belgium) and CTVox (V. 3.2.0 r1294, Bruker microCT, Kontich, Belgium) software.

SEM images of dehydrated and gold-sputtered single bark fibers and fiber pairs of *S. giganteum* were obtained from a Leo 435VP (LEO Electron Microscopy Ltd., Cambridge, England) at 15 kV. The single bark fibers and fiber pairs were extracted in the same way as described later on in Section 4.5 ‘Microtensile Tests’.

Bark samples of *S. giganteum* used to prepare thin sections were dehydrated, embedded in Technovit 7100 and stained with toluidine blue after cutting with a microtome.

### 4.3. Dynamic Drop-Weight Tests

Dynamic drop-weight tests were performed on ten dried bark samples (DIN Norm EN 13183-1) of *S. giganteum* and eight dried bark samples of *A. altissima*. Prior to testing, bark samples were cut to a quadratic basal area of 3 × 3 cm. The sample height was left unaltered and was determined by the bark thickness. The drop-weight test rig was constituted of an instrumented anvil equipped with a 20 kN force sensor (model 8402–6020, Burster Präzisionsmesstechnik GmbH & Co KG, Gernsbach, Germany). After placing a bark sample on top of the instrumented anvil, a drop weight with a mass of 200 g was dropped on to the bark sample from a height of 1.99 m through a guided tube. Regardless of the sample size, the same drop weight and drop height were always used. Drop height and drop weight were determined in preliminary tests, so that a suitable impact and compaction were achieved. The drop-weight tests were recorded with a high-speed camera (model MotionPro Y4, Integrated Design Tools, Inc., Tallahassee, FL, USA). For further details on the test setup, we refer to [16,22].

With the help of the high-speed videos, the velocity of the impactor at any time of the experiment could be determined (by tracking marks that were painted onto the impactor, with the software Motion Studio, IDT Motion Studio, version 2.12.09.04). Converting the velocity of the impactor into its kinetic energy immediately before and after impact allowed the calculation of the energy dissipated during each test. However, it cannot be excluded that a (very) small percentage of this dissipated energy is lost elsewhere in the system (i.e., not by the bark samples), causing a systematic error. Nevertheless, the data derived from the dynamic drop-weight tests allow for a good first-order approximation of the energy dissipated by the tree bark.

Furthermore, the dissipated energy of each sample was converted to an energy density by dividing it by the volume of that sample. This way, the dissipated energy density can be compared with the dissipated energy densities obtained during the quasistatic cyclic compression tests with unchanged maximum strain (see Section 4.4 and Section 2.3.2 Subheading: Cyclic Test with Unchanged Maximum Strain).

### 4.4. Quasistatic Compression Tests

Uniaxial quasistatic compression tests comprising bark samples of *S. giganteum* only were performed using a universal testing machine (Instron 4466-10kN, with a retrofit kit to inspect-DC standard, Hegewald & Peschke Mess- und Prüftechnik GmbH, Nossen, Germany), equipped with a 1 or 10 kN load cell, depending on the maximum force to be expected. LabMaster 2.1.11.4 software (Hegewald & Peschke Mess- und Prüftechnik GmbH, Nossen, Germany) was used to control the universal testing machine. All data were recorded at a sampling rate of 50 Hz.

The sample dimensions were similar to those of the dynamic drop-weight tests, with a basal area of 3 × 3 cm, and the sample height was determined by bark thickness. Depending on which test was performed, several of four predetermined strain rates were applied: 0.01, 0.1, 1 and 10.8 min^−1^. Fixed strain rates were chosen in order to cope with the different sample heights—however, as a consequence, test velocities differ from sample to sample.

Quasistatic compression tests comprised tests including exclusively a loading stage (in the following termed as ‘quasistatic compression tests (one loading stage)’) as well as tests including several cycles, each involving a loading and an unloading stage (in the following termed as ‘cyclic compression tests’).

Quasistatic compression tests (one loading stage) were performed on ten bark samples for each of the four strain rates. These tests were conducted far beyond the yield point, when the densification regime was already reached (until 70% or 80% strain, depending on the strain rate). These tests were carried out to give an overall impression on the bark samples’ behavior during quasistatic compression. Young’s moduli of the samples were calculated using two different approaches: one in which the Young’s modulus was calculated by the maximum slope of each stress–strain curve until 20% strain and one in which the Young’s modulus was calculated with the slope of the best-fit line between 15% and 20% strain (see also Section 2.3, Figure 7 and Figure 8). It needs to be denoted that the term ‘Young’s modulus’ is used in this paper to describe the mechanical property of the bark, which represents a structure and not a homogeneous material. It can thus rather be characterized as a ‘structural’ or ‘apparent’ Young’s modulus. All stresses given for both the quasistatic compression tests and the microtensile tests are nominal (engineering) stresses, and all strains are nominal (engineering) strains.

**Figure 16:**
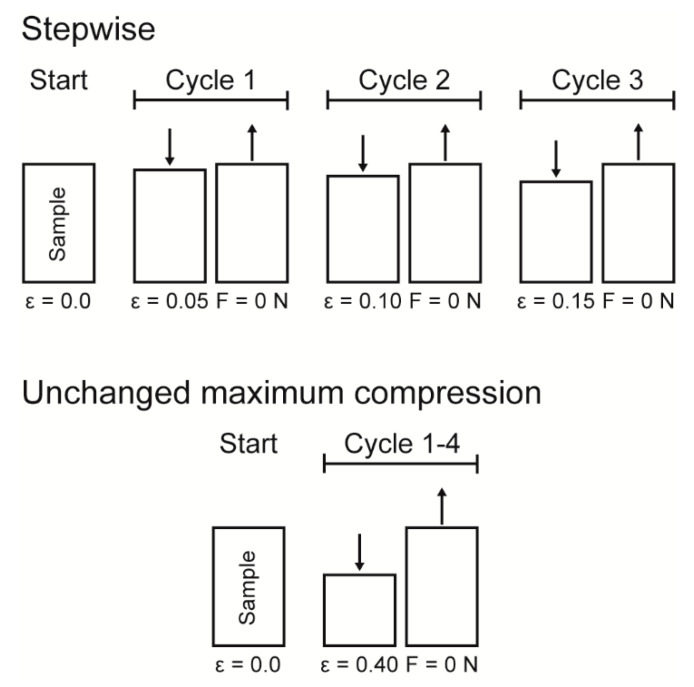
Sketch of the experimental procedure of the quasistatic stepwise cyclic compressions tests and of the quasistatic cyclic compression tests with unchanged maximum compression. During the stepwise cyclic tests, three compression cycles were performed on each sample: first the sample was compressed until 5% strain, then the traverse was lifted until a force of 0 N was reached. The second and the third cycles were performed in a similar manner, only with a different maximum strain (10% and 15%, respectively). During the cyclic tests with unchanged maximum compression, four cycles were performed, each with the same procedure: the sample was compressed until 40% strain, then the traverse was lifted until a force of 0 N was reached.

Cyclic compression tests were performed to gain insights into the potential presence of an elastic, plastic or even viscoelastic deformation behavior and for understanding the energy dissipation. Thereby, two different experimental procedures were carried out for the cyclic tests (see Figure 16 for a sketch of both experimental procedures): (1) a cyclic test with stepwise increasing maximum strain at the end of each cycle and (2) a cyclic test with an unchanged maximum strain at the end of each cycle. The cyclic tests with stepwise increasing maximum strain were conducted in order to characterize the bark samples step by step during rather gentle loading, whereas the cyclic tests with an unchanged maximum strain characterize the durability of the bark under higher loading. For both experimental procedures, ten bark samples for each strain rate (0.01, 0.1 and 1 min^−1^) were tested.

Data obtained from the cyclic compression tests were the residual strains after each cycle and—only for the cyclic test with unchanged maximum strain—energies absorbed/released/dissipated (absorbed energy E_a_, released energy E_r_ and dissipated energy E_d_, in form of energy densities ED). The absorbed energy E_a_ characterizes the energy absorbed in the bark during loading, the released energy E_r_ characterizes the energy released during unloading, and the dissipated energy E_d_ is represented by the difference between these two. Normalizing these energies by the volume of each sample yields the energy density ED, facilitating the comparability to other materials and testing methods (e.g., the dynamic drop-weight tests). The energy density can directly be derived from the stress–strain curves by calculating the area under the respective part of stress–strain curves. The residual strain is calculated for each cycle and is defined as the strain at which a force of 0 N is reached at the end of each cycle.

### 4.5. Microtensile Tests

Microtensile tests were performed on single fibers and fiber pairs extracted from indoor-dried inner and outer bark of *S. giganteum*. These experiments give quantitative insights into the mechanical properties of the basic ‘structural building blocks’ that the bark of *S. giganteum* is composed of. Thus, they provide the basis for the discussion and interpretation of the overall mechanical properties of the bark related to impact damping.

Single fibers and fiber pairs were extracted from the outer and inner bark using tweezers, as suggested by [64] for wood fiber isolation. The cross-sectional dimensions of the fibers and fiber pairs were measured under a microscope (Olympus BX61) at several positions along the fiber(s). Each end of the fiber or fiber pair was glued onto a metal plate with instant glue (Uhu Sekundenkleber blitzschnell mini), followed by mounting the metal plates to the microtensile tester. During this procedure, an adhesive tape fixed the two metal plates in a given position, avoiding loading of the fibers prior to testing. Immediately before the testing procedure, the supporting adhesive tape was cut through. The tensile tests were performed using a microtensile tester (Guangzhou Lok Shun CNC Equipment Ltd., Guanghzou, China) equipped with a 10 N force transducer and controlled with the software LabVIEW (A3M Ingenieursgesellschaft mbH, Braunschweig, Germany). The test velocity was set to 5 µm/s, resulting in strain rates between 0.03 and 0.26 min^−1^.

Due to possible pre-stresses that might occur despite the abovementioned gentle sample mounting procedure involving adhesive tape, the point of zero strain needed to be re-evaluated. Therefore, the best-fit line of the initial linear range (as used also for the calculation of the Young’s modulus) was determined. The intersection of this best-fit line with the abscissa corresponds to the strain at which the fiber is scarcely tensed and was thus defined as zero strain. Two examples of this re-evaluation of the zero strain can be seen in the exemplary stress–strain curves for single fibers and fiber pairs (Figure 14). The tensile strength, maximum strain (i.e., strain of the last data point before sample failure), Young’s modulus and dissipated energy (area under the stress–strain curve from zero strain until maximum strain) were calculated from the stress–strain curves. The sample numbers for the analysis of the tensile strength and the Young’s modulus were nine samples for single fibers of the outer bark, ten for single fibers of the inner bark, eight for fiber pairs of the inner bark and eight for fiber pairs of the outer bark. For the calculation of the maximum strain and the dissipated energy, eight samples for single fibers of the outer bark, seven for single fibers of the inner bark, five for fiber pairs of the inner bark and six for fiber pairs of the outer bark were analyzed.

### 4.6. Statistics

Statistical tests were performed using the R 3.4.0 software (R Core Team 2017 [65]). These tests comprised Mann–Whitney U-tests or Kruskal–Wallis tests (kruskal.test function) followed by post hoc tests by pairwise comparison using the Wilcoxon test (pairwise.wilcox.test function) with Holm adjustment. The statistical tests that were performed are indicated in the caption of each figure. Significant differences—if present—are marked with asterisks in the graphs, with the significance levels indicated as follows: * *p* < 0.05, ** *p* < 0.01, *** *p* < 0.001.

## 5. Conclusions

The bark of the giant sequoia, especially the thick outer bark, is characterized by a high fiber content. These fibers almost entirely consist of cell wall material, have a nearly rectangular cross-section and are laterally connected with adjacent fiber-to-fiber pairs, thus forming a first order of layers. On a second, higher, hierarchical order, layers are not exclusively parallel to the bark surface, but intersect each other, thus forming a three-dimensional network. The compaction behavior of the bark resembles in many ways that of elastomeric open-pore foams. Viscoelastic (most probably material and/or structure immanent, not based on liquid dislocation/squeezing) and elastic deformation components were shown. Furthermore, at least a small plastic component is present, as the bark does not entirely restore its initial shape after compression.

When compared to other tree species, such as *Ailanthus altissima*, the bark of the giant sequoia is capable of dissipating high amounts of impact energy and may thus protect the underlying vital tissues during rockfall events occurring in the natural habitat of the species. Thereby the energy dissipation capability of the bark is even underestimated in the present studies, since the bark structure (interwoven layers of long fibers) is harmed due to cutting of the test samples. The energy dissipation capability becomes even more impressive when keeping in mind that—apart from impact protection—the bark has to fulfill a multitude of different functions and needs to be optimized in terms of multiple criteria towards all of them. The amount of dissipated energy is considerably higher during the dynamic tests compared to quasistatic tests. Thus, due to the marked high strain-rate dependency, the bark of the giant sequoia can be considered as adapted to the naturally occurring conditions during high-energy, high-velocity impact events.

The composition of the bark, especially the subdivision into outer and inner bark, as well as the high fiber content and the arrangement of laterally connected fibers in layers within the outer bark are advantageous during compression. No weakening effect due to degradation, wear or abrasion of fibers was observed for the outer bark compared to the inner bark. Instead, the protective role of the thick and loose outer bark is at least equal to, if not larger than, that of the more compact inner bark. Thus, the outer bark dissipates high amounts of impact energy and reduces force peaks, whereas the inner bark serves as a padding layer. The fibrous and layered bark composition with a huge amount of air-filled spaces between the fibers is also advantageous after an impact event. It facilitates a quick recovery of the bark towards its original structure after compression and thus promotes a quick recovery of the protective function of the bark. In summary, at least on all hierarchical levels examined in this paper (i.e., from the cellular level to the integral level), the bark composition is optimized for energy dissipation under high-velocity impacts. It consists of strong but flexibly interconnected fibers that are arranged in partially interwoven layers on two orders.

All these properties of the giant sequoia bark may serve as a basis for a transfer into bio-inspired fibrous concrete and ceramic components with an increased impact resistance and energy dissipation behavior.

## Figures and Tables

**Table 1:** Mechanical properties (tensile strength, maximum strain and Young’s modulus) of single fibers and fiber pairs of the outer and inner bark of the giant sequoia (median values) compared to literature values of other natural and technical fibers.

Fiber	Tensile Strength [GPa]	Maximum Strain [-]	Young’s Modulus [GPa]
Giant sequoia (outer bark)—single fibers	0.39	0.055	11.1
Giant sequoia (outer bark)—fiber pairs	0.73	0.045	23.2
Giant sequoia (inner bark)—single fibers	0.48	0.027	25.6
Giant sequoia (inner bark)—fiber pairs	1.14	0.025	51.1
Jute (*Corchorus capsularis*)—fiber bundle [38,39,40,41,42]	0.11–0.63	0.015–0.018	10.0–31.2
Hemp (*Cannabis sativa*)—single fiber/fiber bundle [38,43,44,45,46,47,48,49]	0.29–0.89	0.008–0.033	14.4–44.5
Flax (*Linum usitatissimum*)—single fiber [38,50,51,52,53,54]	0.59–1.51	0.016–0.036	37.2–75.1
Ramie (*Boehmeria nivea*)—single fiber [38,55,56,57]	0.56–0.90	0.012–0.025	24.5–65.0
Sisal (*Agave sisalana*)—fiber bundle [38,47,58,59,60]	0.35–0.58	0.023–0.054	9.0–25.0
Cotton (*Gossypium sp.*)—single fiber [38,39,61]	0.29–0.80	0.030–0.100	5.5–13.0
Lyocell [62,63]	0.82		8.6
Nylon [19]	0.45		

## Supplementary Materials

Supplementary materials can be found at https://www.mdpi.com/1422-0067/21/9/3355/s1.

## Abbreviations

E_a_	Absorbed energy
E_r_	Released energy
E_d_	Dissipated energy
ED	Energy density

## Appendix A

This appendix concerns the two approaches for calculating the dissipated energy densities of the dynamic drop-weight tests. For calculating the dissipated energy densities of the dynamic drop-weight tests, two approaches were used concerning which volume to consider when converting the dissipated energy into the dissipated energy density. This was inevitable, as the basal area of the impactor (circular) is not congruent with the basal area of the samples (quadratic). A gentle preparation procedure of the samples involved sawing the sample sides with a Japanese saw instead of using, e.g., a hole saw, which would have introduced high loads to the sample or even destroyed it. This sample preparation resulted in a quadratic basal area of the sample (3 × 3 cm), which gives the best possible congruence with the circular basal area of the impactor (diameter 3 cm). Nevertheless, overhanging corners are inevitable. When considering only the compressed bark volume, i.e., the circular basal area of the impactor, for the calculation of the dissipated energy density, these overhanging corners are disregarded. However, the bark structure involving fibers arranged in interwoven layers results in a partial redirection of the load occurring during an impact event. Thus, not only the bark volume immediately underneath the impactor is involved in energy dissipation, as also shown for plastic foams [18]. Therefore, in a second approach the whole volume of each sample was considered when converting the dissipated energy into the dissipated energy density. Notably, we most likely underestimate the energy dissipation capability of the bark in both approaches by confining the bark samples to the chosen quadratic basal area. If the structure of the bark was still intact covering the entire stem surface, a considerably larger bark volume would be involved in energy dissipation and an even higher amount of dissipated energy could be expected.

## Appendix D

This appendix concerns the differences in strain rates between the quasistatic compression tests and the dynamic drop-weight tests. The strain rate of the dynamic drop-weight tests can only be adjusted by approximation and indirectly by adjusting the drop height of the impactor. However, the velocity of the impactor can be calculated after each test by tracking the impactor in the high-speed videos and then can be converted to a strain rate. Hence, the velocity of the impactor is the basis for calculating not only the energy dissipated during a test, but also the strain rate in the test. This becomes important when comparing the results of the dynamic drop-weight tests with those of the quasistatic compression tests for *S. giganteum* (see Section 2.3). However, the strain rate during a dynamic drop-weight test is not constant like that of a quasistatic compression test. It is highest at the moment when the impactor hits the sample, then continuously decreases to 0 when the sample is maximally compressed and finally gets inverted as the impactor rebounds. Calculating the maximum strain rate (i.e., just when the impactor hits the sample) as well as the medium strain rate during the loading phase (i.e., its mean value derived from the impactor velocities of all high-speed video frames during the time period when the impactor compresses the sample until the sample is maximally compressed) renders the range of the strain rates of the dynamic drop-weight tests, relevant for our considerations. Their mean values amount to 12,238 ± 857 min^−1^ for the maximum strain rate and 7522 ± 602 min^−1^ for the mean strain rate during the loading phase. Thus, if not considering the negligibly short time span shortly before the velocity of the impactor reaches 0, they are in a range much higher than the constant strain rates of the quasistatic compression tests (0.01, 0.1 and 1 min^−1^).
